# Effect of Obesity on Clinical Outcomes in COVID-19 Patients

**DOI:** 10.7759/cureus.33734

**Published:** 2023-01-13

**Authors:** Yahya Habis, Rahmah Alsilmi, Layal Alirbidi, Maha Safhi, Fahad Alsallum, Roaa Alharbi, Abeer Samman

**Affiliations:** 1 Division of Pulmonology, Department of Medicine, Faculty of Medicine, King Abdulaziz University, Jeddah, SAU; 2 Department of Medicine and Surgery, Faculty of Medicine, King Abdulaziz University, Rabigh, SAU; 3 Department of Medicine, King Abdulaziz University Hospital, Jeddah, SAU; 4 Department of Medicine, King Faisal Specialist Hospital and Research Centre, Jeddah, SAU; 5 Department of Medicine, East Jeddah General Hospital, Jeddah, SAU

**Keywords:** infection, hospitalization, icu, covid-19, obesity

## Abstract

Background

Obesity is a well-known risk factor for developing severe coronavirus disease 2019 (COVID-19). In this study, we sought to determine the relationship between obesity and poor outcomes in patients with COVID-19 patients at King Abdulaziz University Hospital (KAUH), Jeddah, Saudi Arabia.

Methods

We conducted a single-centered descriptive study of adult COVID-19 patients hospitalized between March 1 and December 31, 2020, at KAUH. Patients were classified according to body mass index (BMI) as overweight (BMI 25-29.9 kg/m^2^) or obese (BMI ≥30 kg/m^2^). The main outcomes were admission to the intensive care unit (ICU), intubation, and death.

Results

Data were analyzed from 300 COVID-19 patients. Most study participants were overweight (61.8%), and 38.2% were obese. The most significant comorbidities were diabetes (46.8%) and hypertension (41.9%). Both hospital mortality (10.4% for obese; 3.8% for overweight, p = 0.021) and intubation rates (34.6% for obese; 22.7% for overweight, p = 0.004) were significantly higher among obese patients than overweight patients. There was no significant difference in terms of ICU admission rate between both groups. However, intubation rates (34.6% for obese; 22.7% for overweight, p = 0.004) and hospital mortality (10.4% for obese; 3.8% for overweight, p = 0.021) were significantly higher among obese patients than overweight patients.

Conclusions

This study aimed to describe the effect of high BMI on the clinical outcome of COVID-19 patients in Saudi Arabia. Obesity is significantly correlated with poor clinical outcomes in COVID-19. It is also associated with higher mortality and the need for mechanical ventilation necessitating intensive care unit admission. Patients with higher BMI should be prioritized in the hospital setting, as they have a higher potential of developing severe COVID-19 complications and sequelae.

## Introduction

The global coronavirus disease 2019 (COVID-19) pandemic was initially discovered in Wuhan, China, in December 2019 and resulted in a substantial pandemic associated with mortality and morbidities [1]. Comorbidities such as cancer, chronic obstructive pulmonary disease, chronic renal disease, history of solid organ transplant, cardiac problems, type 2 diabetes, and obesity can lead to poor clinical outcomes in the event of developing COVID-19 [2,3].

Obesity was associated with a significantly higher risk of severe pneumonia in hospitalized patients with COVID-19 [4]. According to meta-analysis data, the proportion of obese patients admitted to hospitals with COVID-19, and subsequent mortality increased substantially compared to non-obese patients [5]. Recent studies from numerous countries reported the severity of COVID-19 illness in obese people of varied racial and cultural backgrounds [6-9]. Studies have also shown that obese patients with COVID-19 are at risk of intensive care unit (ICU) admission and mechanical ventilation for respiratory support [10,11]. Obesity in Saudi Arabia has an estimated prevalence of 28.7% of the population [12]. We conducted this retrospective study to review the relationship between obesity and adverse outcomes in our institute and compare our results to previous studies. This study aimed to describe the effect of high body mass index (BMI) on the clinical outcome of patients with COVID-19 infection among Saudi patients.

## Materials and methods

We conducted a single-centered, retrospective descriptive study at King Abdulaziz University Hospital (KAUH), Jeddah, Saudi Arabia. After calculating the sample size using the Raosoft sample size calculator (Raosoft, Inc, Seattle, WA). We included 300 obese and overweight COVID-19 patients, aged 18 or older who were admitted to KAUH between March 1 and December 31, 2020. The study excluded any patients younger than 18, pregnant, had a BMI < 25 kg/m^2^, received only outpatient care, or tested negative for COVID-19. Patient samples to confirm COVID-19 were collected from the upper respiratory tract using a nasopharyngeal swab or the lower respiratory tract by endotracheal aspirate. After acquiring the sample, the reverse transcription-polymerase chain reaction confirmed the diagnosis. We collected data using patient medical records with no contact or interaction with participants. The study was a noninterventional observational study involving a review of medical records, so the requirement for written informed consent was waived. The study was approved by the biomedical research unit of KAUH, Jeddah, Saudi Arabia (Reference No. 516-21).

Data collection method

We recorded patient demographic and anthropometric data and comorbidities. Patients were classified according to BMI as overweight (BMI 25-29.9 kg/m^2^) or obese (BMI > 30 kg/m^2^) according to World Health Organization guidelines [13]. The main outcomes were admission to the ICU, intubation, and death. Additional variables included length of hospital stay, re-admission, hospital-acquired infection (defined as having a positive culture during one admission period), re-intubation, noninvasive ventilation, and treatments (such as therapeutic anticoagulation, tocilizumab, corticosteroids, and plasma exchange).

Statistical analysis

The statistical analysis was performed using RStudio (R version 4.1.1). Descriptive statistics were used for categorical data (frequencies and percentages) and numerical data (means and standard deviation). The differences between BMI groups (overweight and obesity) were tested using a Chi-squared test or Fisher’s exact test for categorical variables. Factors associated with the primary outcomes were assessed by a univariate logistic regression analysis using the primary outcome variable as a dependent variable (intubation, ICU admission, or death) and the demographic variables and comorbidities as independent variables (each in a separate univariate model). In the instance of indicating multiple associations with the independent variables, we constructed a multivariate binary logistic regression model to assess the independent predictors of the outcome variables. Data were expressed as odds ratios (ORs) and 95% confidence intervals (95% CIs). Statistical significance was considered at p < 0.05.

## Results

Demographic characteristics and clinical history of patients

Data were retrieved from 300 patients with a confirmed COVID-19 infection. Approximately two-thirds of them were males (63.3%) and patients aged ≥ 45 years represented 73.4% of the sample. Based on the BMI categories, less than half of the patients were obese (38.3%), whereas overweight patients represented 61.7% of the participants. The proportion of females in the obese group (49.6%) was significantly higher than those in the overweight group (28.6%, p < 0.001). Additionally, the proportion of obese patients among older adults was significantly higher than their overweight counterparts (44.3% vs 31.9%, p = 0.036) (Table 1).

**Table 1:** The association between demographic characteristics and BMI categories Abbreviation: BMI, body mass index.

Parameter	Category	Overall, N = 300	Overweight, N = 185	Obese, N = 115	p-value
Age	18 to <30	17 (5.7%)	15 (8.1%)	2 (1.7%)	0.036
	30 to <45	63 (21.0%)	40 (21.6%)	23 (20.0%)	
	45 to <60	110 (36.7%)	71 (38.4%)	39 (33.9%)	
	60 or more	110 (36.7%)	59 (31.9%)	51 (44.3%)	
Gender	Male	190 (63.3%)	132 (71.4%)	58 (50.4%)	<0.001
	Female	110 (36.7%)	53 (28.6%)	57 (49.6%)	

**Figure 1:**
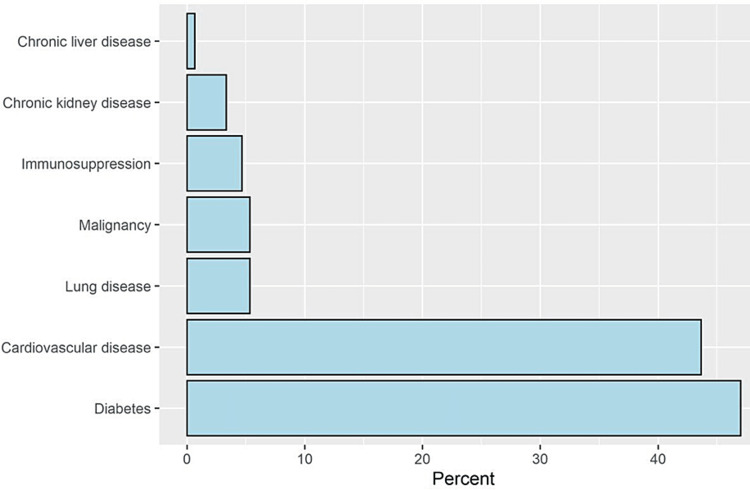
A bar chart depicting the percentages of comorbidities among patients under study.

The most commonly reported comorbidities were diabetes mellitus (47.0%) and cardiovascular diseases (43.7%, Figure 1).

Obesity was significantly associated with having a history of cardiovascular disease (54.8% among obese patients vs 36.8% among overweight, p = 0.002) and a history of chronic kidney disease (7.0% among obese patients vs 1.1% among overweight, p = 0.008, Table 2).

**Table 2:** The association between comorbidities and BMI categories *Immunocompromised: patients having a disease that is associated with a decrease/alteration of the immune system Abbreviation: BMI, body mass index; COPD, chronic obstructive pulmonary disease; ILD, interstitial lung disease; VTE, venous thromboembolism.

Parameter	Overall, N = 300	Overweight, N= 185	Obese, N = 115	p-value
Cardiovascular disease	131 (43.7%)	68 (36.8%)	63 (54.8%)	0.002
Diabetes mellitus	141 (47.0%)	81 (43.8%)	60 (52.2%)	0.157
Chronic kidney disease	10 (3.3%)	2 (1.1%)	8 (7.0%)	0.008
Lung disease	16 (5.4%)	9 (4.9%)	7 (6.1%)	0.634
Chronic liver disease	2 (0.7%)	2 (1.1%)	0 (0.0%)	0.526
Malignancy	16 (5.3%)	11 (5.9%)	5 (4.3%)	0.549
Immunosuppression	14 (4.7%)	9 (4.9%)	5 (4.3%)	0.836

The association between hospital complications and both BMI categories

Regarding the characteristics of hospitalization, there were no significant differences between overweight and obese patients in terms of the rate of ICU admission. However, the proportion of obese patients who required intubation (11.6%) was significantly higher than those in the overweight group (2.7%, p = 0.002). Additionally, the proportion of dead patients in the obese group was significantly higher than their counterparts in the overweight groups (10.4% and 3.8%, respectively, p = 0.021, Table 3).

**Table 3:** The association between hospital complications and BMI categories *Variables were expressed as mean ± standard deviation; otherwise, frequencies and percentages were used to present variables. Abbreviations: BMI, body mass index; ICU, Intensive Care Unit.

Characteristic	Overall, n = 300, n (%)	Overweight, n = 185, n (%)	Obese, n = 115, n (%)	p-value
ICU admission	52 (17.3%)	32 (17.3%)	20 (17.4%)	0.983
Intubation	18 (6.1%)	5 (2.7%)	13 (11.6%)	0.002
Death	19 (6.3%)	7 (3.8%)	12 (10.4%)	0.021

Factors associated with the primary outcomes

Results of the univariate regression analysis showed that intubation was significantly associated with being obese (OR = 4.7, 95% CI, 1.7 to 15.0, p = 0.004). Additionally, ICU admission was significantly higher among males compared to females (OR = 2.8, 95% CI, 1.4 to 6.2, p = 0.005, Table 4). 

**Table 4:** Results of the univariate regression analysis of the factors associated with intubation and ICU admission among patients under investigation. NA: non-available computations because the variable had at least one zero frequency in one group. OR: odds ratio; CI: confidence interval; ICU: intensive care unit

Parameter	Category	Intubation			ICU admission		
		OR	95% CI	p-value	OR	95% CI	p-value
Age	60 or more	—	—		—	—	
	45 to <60	0.53	0.18, 1.45	0.227	0.84	0.43, 1.64	0.610
	30 to <45	0.14	0.01, 0.77	0.066	0.47	0.18, 1.13	0.107
	18 to <30	NA	NA	NA	0.5	0.08, 1.96	NA
Nationality	Non-Saudi	—	—		—	—	
	Saudi	0.84	0.26, 2.32	0.755	0.48	0.22, 0.97	0.053
Gender	Female	—	—		—	—	
	Male	0.9	0.34, 2.50	0.827	2.84	1.41, 6.22	0.005
BMI Group	Overweight	—	—		—	—	
	Obese	4.70	1.72, 15.0	0.004	1.01	0.54, 1.85	0.983
Cardiovascular disease	No	—	—		—	—	
	Yes	1.30	0.49, 3.42	0.593	1.24	0.68, 2.26	0.481
Diabetes mellitus	No	—	—		—	—	
	Yes	0.71	0.26, 1.87	0.499	1.53	0.84, 2.82	0.165
Chronic kidney disease	No	—	—		—	—	
	Yes	1.99	0.10, 11.8	0.529	2.11	0.44, 7.87	0.292
Lung disease	No	—	—		—	—	
	Yes	1.11	0.06, 6.03	0.925	0.67	0.10, 2.48	0.598
Chronic liver disease	No	—	—		—	—	
	Yes	NA	NA	NA	NA	NA	NA
Malignancy	No	—	—		—	—	
	Yes	2.36	0.35, 9.45	0.283	0.67	0.10, 2.49	0.602
Immunosuppression	No	—	—		—	—	
	Yes	1.20	0.06, 6.59	0.865	0.35	0.02, 1.84	0.323

Since only one variable was significantly associated with intubation and ICU admission (one variable for each outcome), constructing a multivariate regression analysis for these outcomes was not applicable. Concerning the factors associated with death, results revealed that death was significantly lower among participants aged 45 to <60 compared to those aged 60 years or older (OR = 0.3, 95% CI, 0.1 to 0.9, p = 0.038). Furthermore, death was significantly higher among obese patients (OR = 3.0, 95% CI, 1.2 to 8.2, p = 0.027), as well as those who were admitted to the ICU (OR = 6.0, 95% CI, 1.6 to 8.0, p < 0.001) and intubated (OR = 5.3, 95% CI, 1.7 to 18.3, p < 0.001) (Table 5). 

**Table 5:** Results of the univariate and multivariate regression analysis of the factors associated with death among patients under investigation. NA: non-available computations because the variable had at least one zero frequency in one group. OR: odds ratio; CI: confidence interval; ICU: intensive care unit

Parameter	Category	Univariate			Multivariate		
		OR	95% CI	p-value	OR	95% CI	p-value
Age	60 or more	—	—		—	—	
	18 to <30	NA	NA	NA	NA	NA	NA
	30 to <45	NA	NA	NA	NA	NA	NA
	45 to <60	0.33	0.10, 0.89	0.038	0.32	0.07, 1.29	0.119
Nationality	Non-Saudi	—	—				
	Saudi	0.8	0.25, 2.15	0.671			
Gender	Female	—	—				
	Male	0.62	0.24, 1.62	0.321			
BMI Group	Overweight	—	—		—	—	
	Obese	2.96	1.15, 8.18	0.027	3.69	0.84, 17.10	0.084
Cardiovascular disease	No	—	—				
	Yes	2.33	0.91, 6.44	0.084			
Diabetes mellitus	No	—	—				
	Yes	1.6	0.63, 4.24	0.329			
Chronic kidney disease	No	—	—				
	Yes	1.68	0.09, 9.69	0.632			
Lung disease	No	—	—				
	Yes	0.98	0.05, 5.29	0.986			
Chronic liver disease	No	—	—				
	Yes	NA	NA	0.990			
Malignancy	No	—	—				
	Yes	3.87	0.83, 13.60	0.050			
Immunosuppression	No	—	—				
	Yes	1.15	0.06, 6.27	0.899			
Intubation	No	—	—		—	—	
	Yes	5.30	1.69, 18.30	<0.001	4.61	0.97, 23.0	0.054
ICU admission	No	—	—		—	—	
	Yes	5.97	1.62, 18.70	<0.001	4.85	1.25, 8.00	<0.001

**Figure 2:**
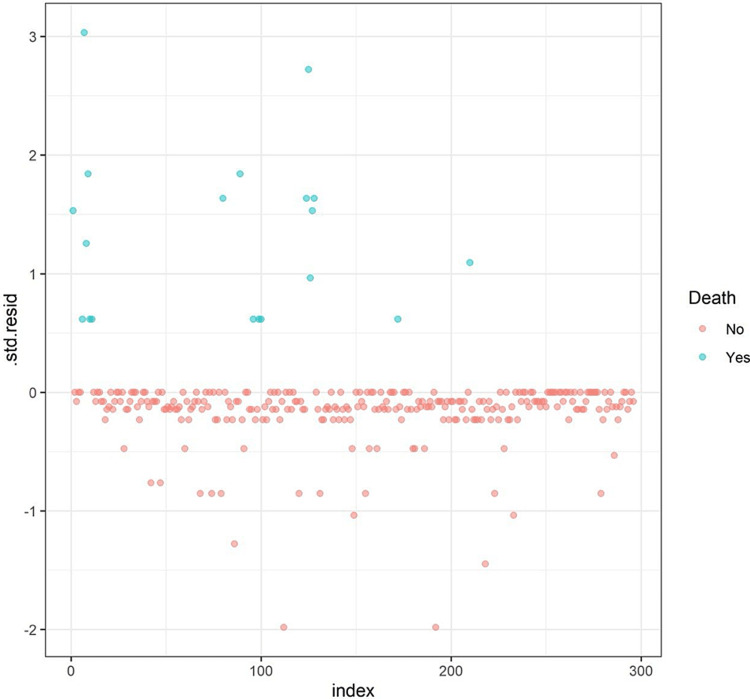
A scatterplot of the standardized residuals of individual data to assess the potential influential data points.

The significantly associated variables were exclusively entered in a multivariate model to account for the independent association between the variables and the risk of death. To fulfill the assumptions of logistic regression, we assessed the risk of multicollinearity using the variance inflation factor (VIF), and we found no risk of multicollinearity (VIF < 5 for all independent variables). Additionally, we excluded one record which was deemed an influential outlier (with the absolute standardized error of >3, Figure 2).

 In the final mode, the independent variables explained 59.8% of the variance in the risk of death. Only ICU admission was an antecedent risk factor for death among patients (OR = 4.9, 95% CI, 1.3 to 8.0, p < 0.001, Table 5).

## Discussion

It is well established that obesity is a clinically important risk factor for severe COVID-19 leading to pneumonia or acute respiratory distress syndrome requiring intubation. Our objective was to investigate the impact of obesity on patients with COVID-19 who required hospitalization, a population particularly vulnerable to serious medical complications [14]. Our results were consistent with several previous reports showing that higher BMI is significantly associated with more severe COVID-19 disease, necessitating hospitalization [15,16].

An earlier study investigating obesity as a risk factor for severe COVID-19 reported that obese patients have altered immune cell activity compared to healthy-weight patients; this alteration of the host defense mechanism puts patients at increased risk of COVID-19 complications [17]. Moreover, a reservoir for viral replication and, therefore, increased viral shedding-can be found in the adipose tissue in obese patients, making them subsequently more susceptible to severe disease courses [18].

Obese patients also have a higher risk of respiratory failure than non-obese patients [19]. Our findings are consistent with previous reports that evaluated the impact of high BMI in hospitalized patients with COVID-19 [20-23]. Our results indicated that the higher the BMI, the higher the risk of intubation, as obese patients required intubation more than overweight patients. This finding could be attributed to a significant decrease in protective cardiopulmonary reserves in obese patients due to adaptation mechanisms in the respiratory system that cause an increase in airway resistance and reduce gas exchange. Further complicating matters, obese patients with COVID-19 have increased immune dysregulation and dysfunction that increase the risk of pneumonia and progression to critical illness and multiorgan failure [24,25].

Furthermore, our study demonstrates that being obese puts the patient at increased risk of death from COVID-19, as it showed that more obese patients died than overweight patients. As previously reported in several cohort studies, obesity is associated with several remarkable comorbidities, such as diabetes, hypertension, heart disease, and kidney disease. However, the mortality estimate in obese patients is directly proportional to the increase in BMI [26,27], which aligns with our results.

This study had some limitations. It included many patients from our institute; however, it is retrospective. Also, data of the included patients were collected before the era of COVID-19 vaccination; therefore, it may not reflect the change in the epidemic noted upon the introduction of the COVID-19 vaccine. Moreover, this study reflects a single-center outcome; the findings cannot be generalized to other Saudi Arabia regions. However, our results are consistent with prior reports from international studies.

## Conclusions

This study aimed to describe the effect of high BMI on the clinical outcome of COVID-19 patients in Saudi Arabia. According to our results, obesity is a significant predictor of the progression of COVID-19 infection to severe pneumonia necessitating mechanical ventilation and death. Clinicians should give special attention to obese COVID-19 patients to improve their survival and disease outcomes.
